# Progress and challenge of microRNA research in immunity

**DOI:** 10.3389/fgene.2014.00178

**Published:** 2014-06-12

**Authors:** Hyang-Mi Lee, Duc T. Nguyen, Li-Fan Lu

**Affiliations:** ^1^Division of Biological Sciences, University of California, San DiegoLa Jolla, CA, USA; ^2^Moores Cancer Center, University of California, San DiegoLa Jolla, CA, USA

**Keywords:** microRNA (miRNA), post-transcriptional regulation, hematopoiesis, immune regulation, immune dysfunction

## Abstract

MicroRNAs (miRNAs) are 19–24 nucleotide long non-coding RNA species that regulate the expression of multiple target genes at the post-transcriptional level. They are required for normal immune system development and function, and their expression is dynamically regulated in different immune cell subsets during lineage differentiation and immune response. Aberrant expression of miRNAs results in dysregulated innate and adaptive immunity. This in turn can lead to failure to fight against invading pathogens and the development of autoimmune diseases and hematopoietic malignancies. In this article, we review current progress in miRNA research in immunity in both physiological and pathological settings. We also discuss research limitations and challenges that researchers are just beginning to solve.

## INTRODUCTION

Gene expression is post-transcriptionally regulated by different types of non-coding RNAs. Among them, microRNAs (miRNAs) inhibit translation or facilitate degradation of target messenger RNAs (mRNAs; Carthew and Sontheimer, 2009). Primary miRNA transcripts are produced by RNA polymerases II and III and processed in the nucleus by the RNase III enzyme Drosha into pre-miRNAs (Lee et al., 2002, 2003, 2004; Yi et al., 2003; Lund et al., 2004; Han et al., 2006). Once shuttled into the cytoplasm, pre-miRNAs are further processed by another RNase III enzyme, Dicer, to produce 19- to 24- base-pair long polynucleotides. These mature miRNAs are incorporated into the RNA-induced silencing complex (RISC), where they interact with the core component protein Argonaute (Ago; Grishok et al., 2001; Hutvagner et al., 2001; Ketting et al., 2001; Hutvagner and Zamore, 2002; Lee et al., 2002; Mourelatos et al., 2002; Lingel et al., 2003). RISC is the functional unit of miRNA-mediated regulation. It uses the “seed sequence” of the miRNA to recognize complementary regions mainly in the 3′ UTRs of mRNAs being targeted for degradation or translational silencing (Hutvagner and Zamore, 2002; Lewis et al., 2005; Pillai et al., 2007). Recent studies have revealed the critical role of miRNAs in tuning immunity. Immune cells express unique miRNA profiles which contribute to their respective functions (Kuchen et al., 2010) and change their miRNA repertoires in response to varying stimuli such as T cell receptor (TCR) activation (Bronevetsky et al., 2013). The past decade has seen many fascinating discoveries about the role of miRNAs in immunity. Unfortunately, the complex natures of the miRNA-mediated gene regulation as well as existing technical challenges have also slowed down research progress.

## CURRENT PROGRESS IN miRNA RESEARCH IN IMMUNITY

### PROGRESS IN FUNCTIONAL ASSESSMENT OF miRNAs IN IMMUNE CELL DEVELOPMENT AND FUNCTION

MicroRNAs were initially discovered for their role in influencing cell fate and differentiation decisions during the development of an organism (Bartel, 2004). In the past decade, mounting evidence has demonstrated that miRNAs are equally important in regulating the immune system. Efforts to discover the cellular and molecular mechanisms of miRNA-mediated immune regulation have relied on gain-of-function and loss-of-function approaches. The general importance of miRNAs in immune cells has been repeatedly confirmed by deletion of key components of the miRNA biogenesis pathway such as Ago, Dicer, and Drosha (Cobb et al., 2005, 2006; Muljo et al., 2005; O’Carroll et al., 2007; Koralov et al., 2008). These gross loss-of-function experiments revealed two important characteristics of miRNA-mediated regulation. First, miRNAs regulate the survival and proliferative function of precursor cells and influence the number and type of differentiated cells that are produced during hematopoiesis and immune responses. In some instances, deletion of all miRNAs greatly promoted one cell type while impairing another. Second, immune cells require miRNAs to carry out their normal functions. Indeed, deletion of Dicer or Drosha in regulatory T (Treg) cells compromised their suppressive capacity and resulted in autoimmune phenotypes (Chong et al., 2008; Liston et al., 2008; Zhou et al., 2008). Taken together, it was reasonable to conclude that miRNA-mediated gene regulation is involved in controlling all aspects of immunity and miRNA dysregulation results in immune-associated phenotypes such as chronic inflammation and autoimmunity by disrupting the normal development, homeostasis, and function of immune cells.

In the past several years, functional studies have further expanded the list of cell types and function regulated by individual miRNAs (**Table 1**). These studies began to discover mechanisms by which miRNAs regulate immune processes. A given miRNA can influence immune cell development by directly inhibiting transcription factors (TFs) and repressors that are crucial for determining cell-type specific differentiation and maintaining lineage identity (Fontana et al., 2007; Johnnidis et al., 2008; Du et al., 2009). miRNAs can also modulate immune responses through targeting key signaling molecules downstream of different immune cell-type specific receptors such as B and T cell receptors as well as innate pathogen recognition receptors (Li et al., 2007; Androulidaki et al., 2009; Hou et al., 2009; O’Connell et al., 2009; Belver et al., 2010; Sheedy et al., 2010). Through repressing the expression of their many targets, miRNAs exert varied, subtle, and often contrasting influence.

**Table 1:** miRNAs involved in immune system.

	Function	miRNA
Innate immunity	Granulocyte development	miR-155, miR-223
	Monocyte development	miR-155, miR-17-92
	Neutrophil function	miR-223
	Macrophage activation	miR-155, miR-146a, miR-21
	Dendritic cell function	miR-155
Adaptive immunity	T cell development	miR-181
	B cell development	miR-150
	T cell proliferation	miR-182, miR-214
	Th1, 17 cell differentiation	miR-155, miR-210, miR-326
	Tfh cell differentiation	miR-10a, miR-17-92
	Treg cell function	miR-155, miR-146a
	CD8 T cell function	miR-155
	B cell function	miR-155, miR-150
Immunological diseases	Autoimmunity	*SLE* miR-23b, miR-146a, miR-125a, miR-21, miR-148a, miR-155, miR-15a
		*RA* miR-23b, miR-146a, miR-155, miR-223
		*MS* miR-155, miR-326, miR-23b, miR-124
	Infectious disease	miR-155
	Immune cell malignancy	miR-15a, miR-16, miR-17-92, miR-155, miR-223, miR-29b

#### miRNA in adaptive immunity

Loss-of-function and gain-of-function studies of individual miRNAs have revealed that a given miRNA can impact different aspects of adaptive immune cell development and function. For example, miR-181 is a positive regulator of B cell differentiation and ectopic expression results in a substantial increase in B cells (Chen et al., 2004). Similarly, miR-17~92 has been shown to be critical in promoting early B cell development, as loss of miR-17~92 leads to increased Bim expression and apoptosis at the pro-B cell to pre-B cell transition (Ventura et al., 2008). In contrast, miR-150 limits early B cell differentiation, since forced expression of miR-150, which targets c-Myb, impaired transition from pro-B cells to pre-B cells (Xiao et al., 2007). In the periphery, several studies have found that miR-155 plays a key role in controlling B cell biology. Mice deficient of miR-155, which is induced during germinal center (GC) reaction *in vivo*, have defects in both antibody secretion and class switching (Thai et al., 2007; Vigorito et al., 2007). The phenotype seems to be a consequence of repressing a number of genes including PU.1 and AID, an enzyme critical for somatic hypermutation and antibody class switching (Vigorito et al., 2007; Teng et al., 2008). In contrast, there is increased follicular B cell activation with enhanced antibody secretion upon T cell-dependent antigen immunization in mice deficient of miR-150 (Xiao et al., 2007).

In T cells, emerging data has suggested that miRNAs also regulate the development and function of different T cell subsets required for adaptive immune response. Loss of miR-155 enhanced Th2 but impaired Th1 and Th17 differentiation (Rodriguez et al., 2007; O’Connell et al., 2010). Th17 differentiation was shown to be regulated by miR-326, too (Du et al., 2009). Recently, attention has also turned to miRNA regulation in follicular T (Tfh) cells, the T cell subset that helps activate B cells during humoral response. Bcl-6, a critical TF for Tfh differentiation, has been shown to regulate expression of several miRNAs (Yu et al., 2009), and miR-10a was known to modulate plasticity of Tfh cells (Takahashi et al., 2012). The latest studies have demonstrated several mechanisms through which miR-17~92 regulates Tfh differentiation that involve targeting RORα and phosphatase, PHLPP2 (Baumjohann et al., 2013; Kang et al., 2013). Similarly, insight into the cell-extrinsic role of miRNAs might be gained from research on Treg cells. It has been reported that miR-155 and miR-146a can influence Treg homeostasis and suppressor function through targeting SOCS1 and Stat1, respectively (Lu et al., 2009, 2010).

#### miRNA in innate immunity

The role of individual miRNAs in controlling the development and function of innate immune cells has also been well documented. For example, miR-17~92 has been shown to be important in monocyte differentiation. miR-17~92 targets Runx1, which promotes monocytopoiesis, while Runx1 suppresses miR-17-92 by binding to its promoter region (Fontana et al., 2007). The range and complexity of miRNA involvement in innate immunity have been further demonstrated through studies of pattern recognition receptor pathways. Toll like receptor (TLR) signaling was discovered to induce expression of a variety of miRNAs including miR-155, miR-146a, and miR-21 (Taganov et al., 2006; O’Connell et al., 2007; Sheedy et al., 2010). In mouse macrophage, miR-155 induced by TLR ligands represses negative regulators of TLR signaling such as SHIP1 and SOCS1 (Androulidaki et al., 2009; O’Connell et al., 2009). In contrast, miR-146a acts as a negative regulator of NF-κB through IRAK1 and TRAF6, and deficiency in miR-146a leads to autoimmunity and myeloid malignancy (Taganov et al., 2006; Boldin et al., 2011; Zhao et al., 2011). The function of miR-146a as a negative regulator of inflammation is also implicated in RIG-I-dependent type 1 interferon (IFN) production by macrophages upon viral infection, in which miR-146a targets IRAK2 (Hou et al., 2009). The discovery of these interactions evidence the dynamism of miRNA activity: inflammatory stimuli can influence miRNA expression, and in turn, individual miRNAs tightly regulate innate immunity by targeting specific mRNAs involved in the activation and resolution of immune responses.

### PROGRESS IN FUNCTIONAL ASSESSMENT OF miRNAs IN IMMUNE DISEASES

Given that miRNAs tune immune cells to function properly, it was not surprising that abnormal miRNA expression leads to immunological disorders. To date, much progress has been made to correlate specific miRNAs with particular disease states, and a large number of miRNAs have been reported to be up- or down-regulated in certain autoimmunity, infectious diseases, and cancers. These aberrant miRNAs have been thought responsible for the inappropriate expression of target proteins associated with the respective pathologies.

#### miRNA in autoimmunity

Recent studies have identified abnormal miRNA expression in many autoimmune diseases including systemic lupus erythematosus (SLE), multiple sclerosis (MS) and rheumatoid arthritis (RA) and the functional relevance of specific miRNAs has been explored in the corresponding mouse models (Junker et al., 2010; Ceribelli et al., 2011; Amarilyo and La Cava, 2012). In human lupus patients, decreased expression of miR-146a leads to hyperactivation of type I IFN and decreased miR-125a to elevated inflammatory chemokine, RANTES (Tang et al., 2009; Zhao et al., 2010). Another set of miRNAs, miR-155 and miR-15a, was found to be increased in the mouse lupus model where Treg cell activity and autoantibody production were affected (Divekar et al., 2010; Yuan et al., 2012). Unlike in lupus, miR-146a is upregulated in RA synovial tissue, where it presumably suppresses proinflammatory cytokines such as TNFα (Nakasa et al., 2008). Similarly, miR-155 and miR-223 are highly expressed in synovial fibroblasts and T cells from RA patients, respectively (Stanczyk et al., 2008; Fulci et al., 2010). Mouse models of arthritis have demonstrated the involvement of miR-155 in regulating B cell and Th17 cell functions attributed to disease development (Kurowska-Stolarska et al., 2011). Among the many miRNAs dysregulated in MS (Junker, 2011), miR-155 was found to modulate astrocyte function in MS and Th17 differentiation in the mouse experimental autoimmune encephalomyelitis (EAE) model (Junker et al., 2009; O’Connell et al., 2010). miR-124 is able to control neuroinflammation by keeping microglia quiescent in steady-state condition; its downregulation at the onset of EAE results in microglia activation and inflammation (Ponomarev et al., 2010). Other autoimmune inflammation including type I diabetes and inflammatory bowel disease (IBD) have been linked to many miRNAs, too (Hezova et al., 2010; Oertli et al., 2011). Nonetheless, the targets of these miRNAs and their mechanisms for regulating autoimmunity remain to be discovered.

#### miRNA in infectious diseases

Several functional studies thus far have demonstrated an important role of miR-155 in host defense against microbial infections. Lack of miR-155 resulted in impaired effector CD8+ T cell function during acute or chronic lymphocytic choriomeningitis virus (LCMV) infection, and defective memory cell differentiation upon infection with *Listeria monocytogenes* (Dudda et al., 2013; Gracias et al., 2013; Lind et al., 2013). Perhaps one of the more surprising discoveries was that miRNAs are directly involved in interactions between host immune responses and infecting pathogens. The fact that viruses themselves generate miRNAs regulating expression of both viral and host genes highlights an essential role of miRNAs in immune responses against infection (Sullivan and Ganem, 2005). Viral miRNA expressed late in SV40 infection down regulates the expression of viral T-antigens in order to avoid alerting cytotoxic T cells (Sullivan et al., 2005). On the other hand, the immune system exerts antiviral activity by regulating the expression of miRNAs in host cells (Pedersen et al., 2007). Some of these host miRNAs were predicted to directly target viral genes. As mediators of host-pathogen interaction, miRNAs influence the outcome of infectious diseases.

#### miRNA in cancer

Differential expression patterns of miRNA have also been found in various malignancies and correlated with clinical outcome (Mi et al., 2007; Marcucci et al., 2008). Studies on the functional relevance of individual miRNAs have revealed that miRNAs can directly modulate the expression levels of oncogenes and tumor suppressor genes, and influence epigenetic regulation, all of which contribute ultimately to tumor development. For example, miRNAs that target anti-apoptotic protein BCL-2 have been found to be deleted in chronic lymphocytic leukemia (CLL; Calin et al., 2002; Cimmino et al., 2005). In mouse lymphocytes, forced expression of miR-17-92 leads to a lymphoproliferative phenotype through targeting tumor suppressive proteins such as PTEN and BIM (Xiao et al., 2008). miR-155 was also found overexpressed in B cell lymphomas, and subsequently shown to target SHIP and C/EBPβ involved in IL-6 signaling (Costinean et al., 2006, 2009). Oncogenic protein can silence the transcription of miR-223 by recruiting chromatin remodeling enzymes (Fazi et al., 2007), while miR-29b promotes expression of tumor suppressor genes by repressing DNA methyltransferases in acute myeloid leukemia (AML; Garzon et al., 2009). Research on the role of miRNAs in immune cell malignancies complemented concurrent research on how miRNAs regulate proliferation and differentiation.

## CURRENT CHALLENGES IN miRNA RESEARCH IN IMMUNITY

MicroRNAs control many important immunological processes, and much like TFs, they exhibit diverse effects through their action on multiple mRNA species. As such, miRNA regulatory networks are complex, and before designing any kind of treatment, the networks must be understood in all their complexities. Despite the great efforts that have been committed to discover the precise role of miRNAs in the immune system, many issues remain unsolved. Some of these are technical limitations of current approaches, such as the sensitivity of *in vivo* and *in vitro* assays, and the ability to isolate sufficient cells of certain immune cell subsets for miRNA profiling and functional analysis. At the same time, the biology of miRNA-mediated regulation also presents inherent difficulties, such as transient low level induction of miRNAs under certain circumstances and the presence of isomiRs (Morin et al., 2008). Here, we will discuss two major challenges that researchers are just beginning to solve.

### CHALLENGE OF miRNA TARGET IDENTIFICATION

In the past decade, many computational algorithms have been developed to identify potential miRNA target genes (Bartel, 2009). With time, performance and target prediction have improved significantly, and different prediction methods now share a high degree of overlap. Advances in computational prediction could be largely attributed to the recognition of the importance of seed pairing (Lewis et al., 2005). Unfortunately, many important functional miRNA targets could not be identified due to the inability of these tools to find miRNA binding sites with seed mismatches (Doran and Strauss, 2007). Moreover, most of the existing miRNA target predictions have been restricted to mRNA 3′ UTRs. Genes that are regulated by miRNA through binding in the 5′ untranslated, promoter or protein coding regions were likely missed (Lytle et al., 2007; Place et al., 2008; Tay et al., 2008). A few years ago, High-throughput sequencing of RNA isolated by crosslinking immunoprecipitation (HITS-CLIP) or photoactivatable-ribonucleoside-enhanced crosslinking and immunoprecipitation (PAR-CLIP) techniques were developed (Chi et al., 2009; Hafner et al., 2010; Zisoulis et al., 2010; Van Wynsberghe et al., 2011). These experimental approaches promised to provide direct biochemical evidence of specific miRNA–mRNA interactions without the false positives and negatives of bioinformatic prediction, but they had their own limitations. Because biochemical identification of Ago binding sites came from HITS analysis of pooled mRNA after Ago immunoprecipitation, there was no easy way to identify the corresponding miRNAs responsible for Ago binding. The difficulty increased when it was discovered that a substantial number of Ago binding sites identified in those studies did not contain clear seed matches. It was uncertain whether this apparently seedless targeting was caused by non-canonical miRNA-target interactions or miRNA-independent mechanisms (Leung et al., 2011). One strategy to overcome the uncertainty was to perform differential HITS-CLIP (dCLIP) analysis and compare mRNA expression changes in T cells with or without a single miRNA; in this case, miR-155 was chosen (Loeb et al., 2012). Combined with luciferase reporter assay and site-directed mutagenesis studies, the authors demonstrated that miR-155 could target and repress genes through binding to sites without canonical seed matching. Finally, since many Ago-interacting proteins have been described in the past and it seems highly unlikely that all of them constitute a common complex (Nilsen, 2007), it is plausible that the cell type of interest or even the differentiation state of the cell could all impact miRNA-mediated gene regulation. Thus, it will be useful to conduct the aforementioned dCLIP analysis with a given miRNA in other immune cell types in a genetically controlled manner to gain clearer understanding of the role of miRNA in immune system.

### CHALLENGE OF FUNCTIONAL VALIDATION OF miRNA TARGETS

**FIGURE 1:**
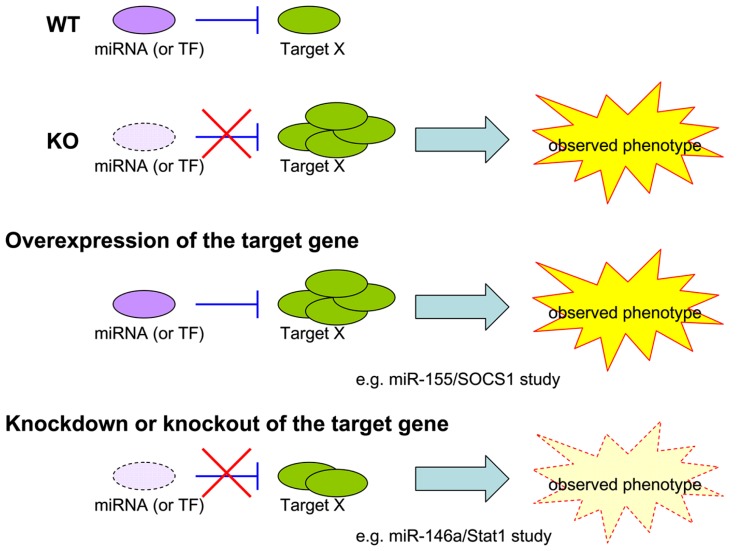
**Models for miRNA study.** Current experimental approaches to validate the biological significance of a single target repressed by a given miRNA in an observed phenotype.

As 100s of genes could be regulated by a single miRNA, its loss often leads to multiple and complicated biological consequences. Because many of the targets could also operate in a collaborative or competitive manner, it is almost impossible to attribute an observed miRNA-dependent phenotype to the regulation of a single target. To resolve this second challenge in miRNA research in immunity, studies heretofore have mostly relied on two genetic approaches: (1) an overepxression approach in which a chosen miRNA-dependent phenotype is recapitulated upon overexpression of a proposed miRNA target in a WT animal [i.e., our miR-155/SOCS1 study in Treg cells (Lu et al., 2009)]; (2) a knockdown/knockout approach in which a selected miRNA-dependent phenotype is rescued by knocking down the proposed miRNA target in the miRNA KO animal [i.e., our miR-146a/Stat1 study in Treg cells (Lu et al., 2010); **Figure 1**]. While both approaches are valid and provide strong correlative support for hypothesized models, neither could faithfully represent the dynamic miRNA-mediated regulation in a given cell type and in a given time *in vivo*, as both miRNAs and their targets are also subjected to regulation in response to different stimulation and environmental cues. The ideal method to address this challenge is to generate a mutant mouse line harboring mutations in the miRNA binding site of the target gene. The introduced mutations should only disrupt the interaction of the selected miRNA with the gene of interest. By comparing these mice to miRNA-deficient mice, one would be able to isolate the effects of a miRNA to one key immune regulator and the biological significance of single target repression in miRNA-mediated immune regulation could be determined. The method is theoretically sound and should provide the most direct experimental evidence of how a single target controlled by one miRNA controls immune responsiveness. To date, however, it has only been taken in two studies, both of which demonstrated a key role of miR-155-mediated regulation of AID in controlling B cell function (Dorsett et al., 2008; Teng et al., 2008). One could argue that the impact of those studies is somewhat limited due to the restricted expression of the target gene, AID, in the B cell lineage. Considering the time and financial commitment needed to generate a mouse model that could only answer such a narrow question, it is not too surprising why this approach has not been applied to more miRNA research. Nevertheless, as the gene targeting techniques [e.g., CRISPR/Cas-mediated genome engineering (Yang et al., 2013)] have been improving rapidly in the last couple years, more miRNA studies using the aforementioned knock-in type of approach should become available in the near future.

## CONCLUDING REMARKS

In the past decade, intensive investigation in miRNA-mediated gene regulation has demonstrated that miRNAs are key regulators of the development and function of the immune system. As such, miRNAs are differentially expressed in immune cell subsets and tightly regulated to ensure homeostasis and proper immune response. Studies in different disease conditions have further revealed their role in immune system dysregulation and pathogenesis. Despite the highly complex nature of miRNA regulatory networks and the existence of aforementioned technical limitations, recent advances have made miRNA biology a fascinating subject in immunological research. From a basic immunological point of view, the control of immune cell biology by miRNAs provides a powerful model to dissect the molecular orchestration of cellular differentiation, function, and homeostasis. On a practical level, manipulating miRNA pathways in immune cells promises to offer novel therapeutic approaches in the treatment of autoimmunity, infectious disease, and immune malignancies.

## Conflict of Interest Statement

The authors declare that the research was conducted in the absence of any commercial or financial relationships that could be construed as a potential conflict of interest.
